# O-GlcNAcylation: A major nutrient/stress sensor that regulates cellular physiology

**DOI:** 10.1016/j.jbc.2024.107635

**Published:** 2024-08-05

**Authors:** Lance Wells, Gerald W. Hart

**Affiliations:** Complex Carbohydrate Research Center, Department of Biochemistry & Molecular Biology, University of Georgia, Athens, Georgia, USA

O-GlcNAcylation of nuclear, cytoplasmic, and mitochondrial proteins was discovered over 4 decades ago (1, 2). Work from many laboratories has shown that the cycling of O-linked N-acetylglucosamine (O-GlcNAc) on proteins serves as an essential nutrient/stress sensor to regulate transcription, signaling, DNA methylation and repair, translation, protein trafficking, and cell division in response to glucose and other nutrients (for review, (3)). Global O-GlcNAcylation is also increased in most forms of cellular stress (4). Currently, there are known to be at least 8000 human proteins modified and regulated by this dynamic sugar modification of proteins (5). O-GlcNAcylation has extensive crosstalk with phosphorylation both at the site level on polypeptides and by modifying many kinases. O-GlcNAcylation also cross-talks to regulate ubiquitination, methylation, acetylation, and other post-translational modifications. The molecular functions of O-GlcNAc depend not only on the protein that is modified but also on the site on the polypeptide to which it is attached. Due to O-GlcNAc’s critical role in regulating nearly every cellular process, it is not surprising that it plays a direct role in the etiology of metabolic diseases associated with aging, including diabetes, cardiovascular disease, cancer, and neurodegeneration (6, 7) (Fig. 1). In this Thematic Series, we have selected topics that first illustrate the state of our knowledge about the roles of O-GlcNAcylation in fundamental cellular processes, such as signaling, transcription, DNA repair, circadian clocks, and the regulation of cell division, and follow with five reviews on O-GlcNAc’s roles in various human diseases.

**Figure 1:**
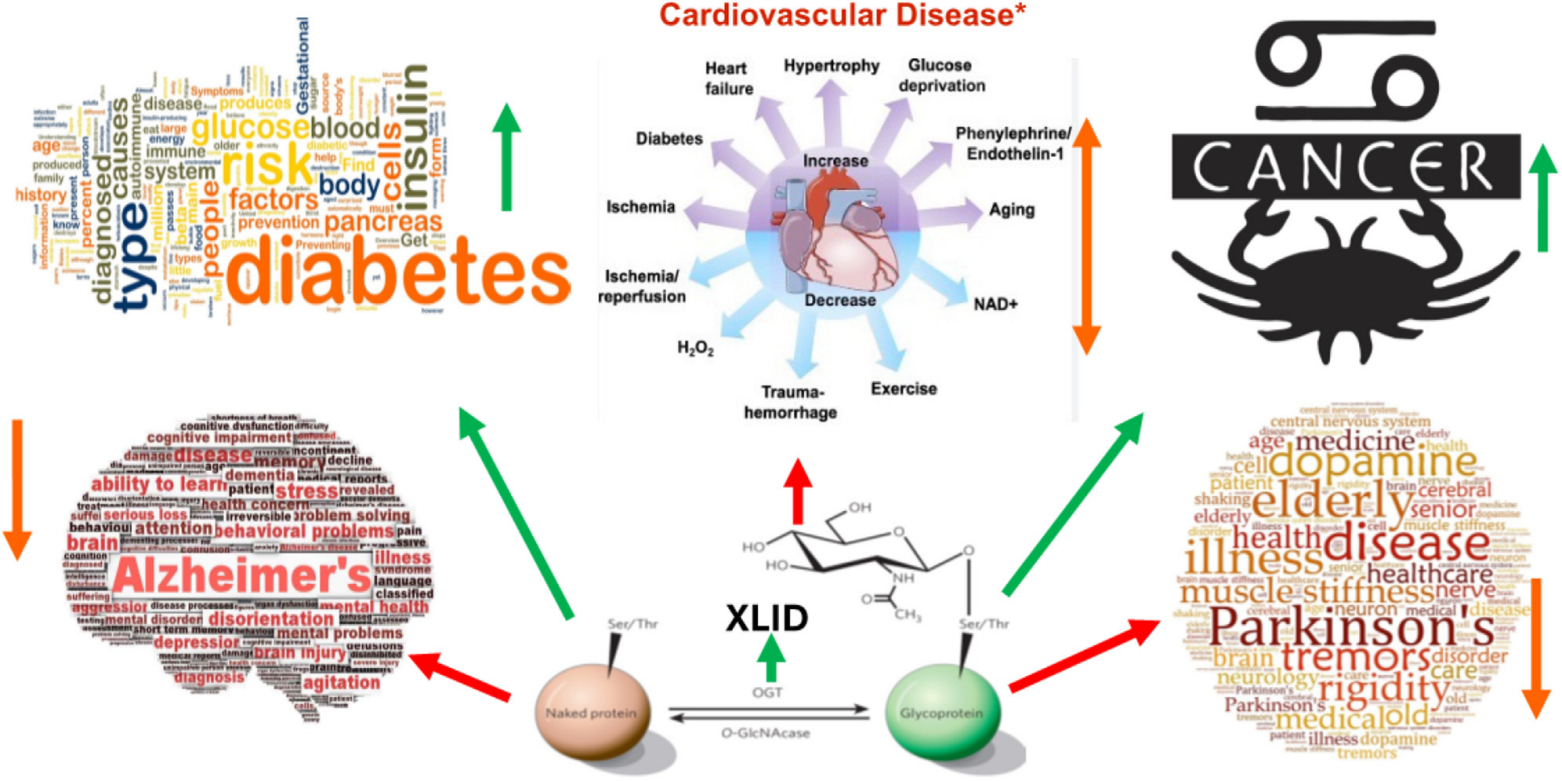
**O-GlcNAc is Involved in Chronic Diseases of Aging.** A PubMed search on May 28, 2024 of “O-GlcNAc and diabetes ” = 475 citations; of “O-GlcNAc and Cardiovascular” = 247 citations; of “GlcNAc and cancer” = 627 citations; of “O-GlcNAc and Alzheimers” = 170 citations; and of “O-GlcNAc and Parkinsons” = 29 citations. Cardiovasular disease portion of the figure was adapted from (20). *Arrows* indicate changes in global O-GlcNAcylation associated with the disease.

Nelson *et al.* (8) present an overview of how O-GlcNAcylation regulates key signaling pathways, including G-protein coupled receptors, growth factors, mitogen-activated protein kinase pathways, lipid sensing, and cytokine signaling pathways. They also summarize methodological and chemical biology advances that have allowed the elucidation of O-GlcNAc’s roles in these signaling pathways. Brian Lewis (9) summarizes the evidence that O-GlcNAcylation of RNA polymerase II and basal transcription factors, plays critical roles in initiation, pausing, and elongation during the transcription cycle. Wu *et al.* (10) review mechanisms by which O-GlcNAcylation regulates various DNA damage response pathways including, homologous recombination, non-homologous end joining, base excision repair, and translesion DNA synthesis. Liu *et al.* (11) discuss the roles of O-GlcNAcylation in the modulation of circadian rhythms by the timing of food intake and conversely how O-GlcNAcylation is regulated by circadian clocks independent of food consumption time. They further propose that O-GlcNAcylation is a generalized cellular status effector that responds to cellular signals and conditions, such as endoplasmic reticulum stress, apoptosis, and infection. Zhang and Wang (12) describe how O-GlcNAcylation of key proteins involved in protein trafficking within cells, such as coat protein complexes (COPI and COPII), clathrin, SNARES, and GRASP55, regulate vesicle budding and fusion in both anterograde and retrograde trafficking. Saunders *et al.* (13) review the body of evidence that O-GlcNAcylation regulates cellular progression through the cell cycle. They highlight O-GlcNAc’s roles in regulating the G_1_ phase of the cell cycle, its control of DNA replication and repair, and its organization of mitotic progression and spindle dynamics.

The final five reviews in this Thematic Series describe the myriad roles that O-GlcNAcylation plays in the etiologies of genetic diseases of development, and chronic diseases associated with aging (Fig. 1). Mayfield *et al.* (14) describe our current knowledge on an O-GlcNAc transferase (OGT) congenital disorder of glycosylation (OGT-CDG) that results in X-linked intellectual disability that were first biochemically characterized in 2017 (15). They propose that different variants in OGT may disrupt the enzyme’s interactome and contribute to the heterogeneous neural-specific phenotypes of OGT-CDGs and dysregulation of neuronal development. Umapathi *et al.* (16) describe the paradoxical roles that O-GlcNAcylation plays in the heart. Short-term elevated O-GlcNAcylation protects the heart from environmental stresses such as heat shock, hypoxia/reoxygenation injury, and sepsis by preventing apoptosis and necrosis. In contrast, prolonged elevation or reduction in O-GlcNAcylation produces a maladaptive response associated with cardiac pathologies, such as hypertrophy and heart failure. Ramakrishnan (17) summarizes the critical roles O-GlcNAcylation plays in the regulation of physiological and pathological aspects of cellular immune functions. In particular, he also enumerates major remaining questions in our understanding of the molecular processes as to how nutrients regulate immunity. Minh *et al.* (18) describe how O-GlcNAcylation is increased in nearly all types of cancer, and how O-GlcNAcylation plays a critical role in regulating many cellular processes that are hallmarks of cancer. Pratt and Vocadlo (19) describe the development of potent and highly specific inhibitors of O-GlcNAcase (OGA), and their potential uses as drugs to treat Alzheimer’s and Parkinson’s disease. They also illustrate how these OGA inhibitors and the development of chemical methods to stoichiometrically O-GlcNAcylate proteins have clarified the effects of O-GlcNAc on protein aggregation and in regulating heat shock proteins.

At the time of its discovery over 40 years ago, it was dogma that protein glycosylation did not occur in the nucleus and cytoplasm. However, we now know that O-GlcNAcylation is not only the most dynamic form of protein glycosylation that serves as a major nutrient/stress sensor to regulate nearly every cellular process but also that dysregulation of O-GlcNAcylation underlies many of the etiologies of major chronic diseases associated with aging (Fig. 1). This special issue illustrates some of the best examples of O-GlcNAc’s myriad roles in cellular physiology and in human disease. Hopefully, these reviews will inspire future research into this rapidly developing field.

## Data availability

The datasets generated during and/or analyzed during the current study are available from the corresponding authors on reasonable request.

## Conflict of interest

The authors declare that they have no conflicts of interest with the contents of this article.
